# Likelihood of aggressive PK/PD target attainment of continuous-infusion beta-lactams during the first week of treatment of febrile neutropenia: findings from a 1-year prospective, monocentric study in onco-haematological patients

**DOI:** 10.1093/jac/dkag183

**Published:** 2026-06-03

**Authors:** Pier Giorgio Cojutti, Alice Toschi, Zeno Pasquini, Stefania Paolini, Enrico Maffini, Francesca Bonifazi, Pier Luigi Zinzani, Maddalena Giannella, Pierluigi Viale, Federico Pea

**Affiliations:** Department of Medical and Surgical Sciences, Alma Mater Studiorum, University of Bologna, Bologna 40138, Italy; Clinical Pharmacology Unit, IRCCS Azienda Ospedaliero-Universitaria di Bologna, Bologna, Italy; Infectious Diseases Unit, IRCCS Azienda Ospedaliero-Universitaria di Bologna, Bologna, Italy; Infectious Diseases Unit, IRCCS Azienda Ospedaliero-Universitaria di Bologna, Bologna, Italy; Institute of Hematology ‘L. and A. Seràgnoli’, IRCCS Azienda Ospedaliero–Universitaria di Bologna, Bologna, Italy; Institute of Hematology ‘L. and A. Seràgnoli’, IRCCS Azienda Ospedaliero–Universitaria di Bologna, Bologna, Italy; Institute of Hematology ‘L. and A. Seràgnoli’, IRCCS Azienda Ospedaliero–Universitaria di Bologna, Bologna, Italy; Department of Medical and Surgical Sciences, Alma Mater Studiorum, University of Bologna, Bologna 40138, Italy; Institute of Hematology ‘L. and A. Seràgnoli’, IRCCS Azienda Ospedaliero–Universitaria di Bologna, Bologna, Italy; Department of Medical and Surgical Sciences, Alma Mater Studiorum, University of Bologna, Bologna 40138, Italy; Infectious Diseases Unit, IRCCS Azienda Ospedaliero-Universitaria di Bologna, Bologna, Italy; Department of Medical and Surgical Sciences, Alma Mater Studiorum, University of Bologna, Bologna 40138, Italy; Infectious Diseases Unit, IRCCS Azienda Ospedaliero-Universitaria di Bologna, Bologna, Italy; Department of Medical and Surgical Sciences, Alma Mater Studiorum, University of Bologna, Bologna 40138, Italy; Clinical Pharmacology Unit, IRCCS Azienda Ospedaliero-Universitaria di Bologna, Bologna, Italy

## Abstract

**Background:**

Onco-haematological patients with febrile neutropenia are at high risk of severe infections caused by multidrug-resistant pathogens. Continuous infusion (CI) of beta-lactams may improve pharmacokinetic/pharmacodynamic (PK/PD) target attainment, yet evidence in this population remains limited.

**Objectives:**

To evaluate the likelihood of achieving an aggressive PK/PD target with CI beta-lactams during the first week of therapy in onco-haematological patients and to identify predictors of target non-attainment.

**Methods:**

This prospective observational study enrolled adult onco-haematological patients receiving CI beta-lactams for empirical or targeted treatment over a 1-year period. Steady-state plasma concentrations were measured between days 3 and 7. The aggressive PK/PD target was defined as a free steady-state concentration-to-MIC (or clinical breakpoint) ratio ≥4; for beta-lactam/beta-lactamase inhibitor combinations, a joint PK/PD target was applied. Multivariate logistic regression analysis identified factors associated with target attainment.

**Results:**

A total of 256 patients were included, 82 of whom (32.1%) received targeted therapy. Aggressive PK/PD target attainment was achieved in 85.4% of patients undergoing targeted treatment, compared with 57.5% of those treated empirically. Target non-attainment occurred most frequently with piperacillin/tazobactam and ceftazidime/avibactam. Augmented renal clearance (OR 12.29, *P* < 0.001), male sex (OR 3.79, *P* < 0.001) and age <65 years (OR 2.40, *P* = 0.025) independently predicted target non-attainment, whereas targeted therapy and meropenem use were associated with a higher attainment.

**Conclusions:**

CI beta-lactams reliably achieve aggressive PK/PD targets during targeted therapy but not during empirical treatment in onco-haematological patients. Augmented renal clearance is a major determinant of underexposure, supporting the use of therapeutic drug monitoring to optimize dosing in this high-risk population.

## Introduction

Onco-haematological patients may be particularly vulnerable to bacterial infections because of severe immunosuppression.^1,2^ Febrile neutropenia (FN) is usually the first and often the only manifestation of a bacterial infection and needs prompt empirical treatment with an antipseudomonal beta-lactam for preventing the potential evolution to sepsis or septic shock.^3,4^ Current guidelines recommend piperacillin/tazobactam, cefepime or ceftazidime as first-line treatment. Meropenem is recommended in patients with known colonization or previous infection with extended spectrum cephalosporin resistant (ESCR) strains, in those with severe infections, or in centres with high prevalence of ESCR *Enterobacterales*.^3,5–7^ Unfortunately, the selective pressure associated with the repeated antibiotic use and the prolonged hospitalizations of onco-haematological patients may promote colonization of these patients by MDR pathogens.^1,2^ In is worth noting that, in recent years, several carbapenem-resistant (CR) Gram-negatives emerged as major pathogens of FN episodes, namely CR-*Enterobacterales* such as KPC- or OXA-48-producing *Klebsiella pneumoniae* or *Escherichia coli* and CR-*Pseudomonas aeruginosa*.^8,9^ FN episodes occurring in onco-haematological patients carrying these CR pathogens are of particular clinical concern and require prompt adequate treatment, as the mortality rates associated with these infections may be as high as 36%–58%.^10,11^ In these cases, international guidelines recommend the use of beta-lactam/beta-lactamase inhibitor combination (BL/BLIc) such as ceftazidime/avibactam, meropenem/vaborbactam or imipenem/relebactam against KPC-producing *Enterobacterales*, ceftazidime/avibactam against OXA48-producing *Enterobacterales* and ceftolozane/tazobactam or ceftazidime/avibactam against MDR-*Pseudomonas aeruginosa*.^7^

All the aforementioned beta-lactams exhibit time-dependent antibacterial activity, and the conventional pharmacokinetic/pharmacodynamic (PK/PD) target associated with clinical efficacy is 40%–100% of time with concentrations persisting above the MIC of the pathogen.^12^ Consequently, the posology chosen in the clinical trials of each of these different agents was essentially developed for meeting this PK/PD target. However, it has been recently shown among critically ill patients having targeted treatment of Gram-negative infections that this conventional target of beta-lactams may not suffice either for maximizing clinical efficacy or for preventing resistance development associated with beta-lactam use.^13,14^ Specifically, a meta-analysis showed that attaining an aggressive PK/PD target (defined as 100% of time with concentrations persisting 4-fold above the MIC of the pathogen) granted a higher clinical cure rate (OR 1.69; 95% CI 1.15–2.49) and was more protective against the risk of beta-lactam resistance development than traditional beta-lactams (OR 0.06; 95% CI 0.01–0.29) when compared with the conventional target.^13,14^ Of note, delivering beta-lactams by continuous infusion (CI) may be worthwhile for maximizing the likelihood of aggressive PK/PD target attainment with beta-lactams under standard dosing regimens.^15,16^ Looking at the novel beta-lactams, a recent pre–post quasi-experimental study explored the impact of aggressive joint pharmacokinetic/pharmacodynamic target attainment with CI ceftazidime/avibactam on treatment outcome of KPC-producing *Klebsiella pneumoniae* infections and on ceftazidime/avibactam resistance development.^17^ Interestingly, it was shown in the post-intervention phase that attaining an aggressive joint PK/PD target was protective against 90-day resistance development to ceftazidime/avibactam (OR 0.07; 95% CI 0.01–0.69).^17^

On the basis of these assumptions, the aim of this prospective study was to assess the likelihood of aggressive PK/PD target attainment during the first week in a cohort of onco-haematological patients receiving empirical or targeted treatment of FN with CI of different beta-lactams, according to clinical needs, and to identify potential clinical factors affecting the attainment.

## Methods

### Study design

This prospective, monocentric, observational study was carried out between January and December 2024 at the Clinic of Hematology of the IRCCS, Azienda Ospedaliero-Universitaria di Bologna, Italy. Enrolled patients received empirical or targeted treatment of FN with different beta-lactams by CI, according to the clinical needs, and underwent therapeutic drug monitoring (TDM) during the first 7 days of treatment for assessing aggressive PK/PD target attainment or non-attainment.

FN was defined as an absolute neutrophil count (ANC) < 0.5 × 10^9^/L or ANC < 1.0 × 10^9^/L predicted to decline to ANC <0.5 × 10^9^/L coupled with an oral temperature of >38°C on two consecutive measurements.^18^ Empirical treatment was usually based on an antipseudomonal beta-lactam monotherapy, started with a loading dose (LD) followed by a maintenance dose (MD) delivered by CI and adjusted by estimated glomerular filtration rate (eGFR) [piperacillin/tazobactam (LD of 4.5 g followed by a MD of 18 g for eGFR >40 mL/min/1.73 m^2^, 13.5 g for eGFR between 20 and 40 mL/min/1.73 m^2^ and 9 g daily for eGFR <20 mL/min/1.73 m^2^) or ceftazidime (LD of 1 g and followed by a MD of 6 g for eGFR >50 mL/min/1.73 m^2^ or 3 g for eGFR ≤50 mL/min/1.73 m^2^)]. In case of known colonization or previous infection with ESCR strains therapy with CI meropenem after loading (LD of 1 g and followed by a MD of 1 g q8h over 8 h if eGFR >60 mL/min/1.73 m^2^ or 0.5 g q6h over 6 h if eGFR <60 mL/min/1.73 m^2^) is started.

In cases of rectal colonization with, previous infections with or of microbiological documented infections caused by CR pathogens, different novel beta-lactams were used on the basis of antimicrobial susceptibility testing [ceftolozane/tazobactam (LD of 2 g/1 g followed by a MD of 6 g/3 g daily by CI if eGFR >50 mL/min/1.73 m^2^, 3 g/1.5 g daily by CI if eGFR between 30 and 50 mL/min/1.73 m^2^, 1.5 g/0.75 g daily by CI if eGFR 15–29 mL/min/1.73 m^2^ and 0.9 g/0.45 g daily by CI if eGFR <15 mL/min/1.73 m^2^), or ceftazidime/avibactam (LD of 2.5 g followed by a MD of 2.5 g q8h if eGFR >50 mL/min/1.73 m^2^, 1.25 g q8h over 8 h if eGFR between 31 and 50 mL/min/1.73 m^2^ and 0.625 g q8h over 8 h if eGFR <31 mL/min/1.73 m^2^) or meropenem/vaborbactam (LD of 2 g/2 g followed by a MD of 2 g/2 g q8h over 8 h if eGFR >50 mL/min/1.73 m^2^ or 1 g/1 g q8h over 8 h if eGFR ≤50 mL/min/1.73 m^2^) or cefiderocol (LD of 2 g followed by a MD of 2 g q6h over 6 h if eGFR >120 mL/min/1.73 m^2^, 2 g q8h over 8 h if eGFR between 60 and 120 mL/min/1.73 m^2^, 1.5 g q8h over 8 h if eGFR 30–59 mL/min/1.73 m^2^, 1 g q8h over 8 h if eGFR 15–29 mL/min/1.73 m^2^)].

Generally, when central venous catheter-related infection was suspected, or on signs of skin and soft tissue infection, or a Gram-positive was isolated by blood cultures, an anti-Gram-positive therapy with vancomycin, daptomycin or linezolid was added.

Patients being at high risk of invasive fungal infections (IFIs) [namely, having at least one of the following: previous IFI in the last 2 years, >7 days of aplasia, a human leucocyte antigen (HLA)-matched unrelated donor (MUD), HSCT or an haploidentical HSCT with graft-versus-host disease (GVHD)] received antifungal prophylaxis with an anti-mould drug, generally a triazole (e.g. posaconazole, voriconazole or isavuconazole).

All patients underwent one TDM instance of beta-lactam steady-state plasma concentration (Css) in the period between days 3 and 7, depending on clinical feasibility. A peripheral venous blood sample (4 mL) was drawn and sent promptly to the laboratory. After centrifugation at 3500 rpm for 5 min, plasma was separated and drug concentrations were analysed by means of validated LC-MS HPLC methods. A commercially available method (Chromsystems Instruments & Chemicals GmbH, Munich, Germany) validated in LC-MS was used for measuring piperacillin/tazobactam, meropenem and ceftazidime,^19^ whereas specifical LC-MS validated methods were used for measuring ceftazidime/avibactam,^20^ ceftolozane/tazobactam,^21^ meropenem/vaborbactam^22^ and cefiderocol.^23^ Precision and accuracy were assessed by replicate analyses of quality control samples against calibration standards. Intra- and inter-assay coefficients of variation were always <10%.

Only total plasma Css were measured and the free fractions (*f*) were calculated considering a plasma protein binding of 30% for piperacillin,^24^ 10% for ceftazidime,^25^ 20% for ceftolozane,^26^ 2% for meropenem,^24^ 58% for cefiderocol,^27^ 7% for avibactam,^25^ 23% for tazobactam^26^ and 33% for vaborbactam.^28^

Aggressive PK/PD target attainment was defined as a beta-lactam *f*Css/MIC ratio or a *f*Css/MIC_BP_ ratio ≥4 (where *f*Css is free steady-state plasma concentration and MIC_BP_ is the EUCAST clinical breakpoint against susceptible isolated pathogens) for targeted or empirical treatment, respectively. When using BL/BLIc, an aggressive joint PK/PD target was defined, as previously reported.^29^ Briefly, aggressive joint PK/PD target was considered attained whenever both a beta-lactam *f*Css/MIC ratio >4 and a beta-lactamase inhibitor (BLI) *f*Css/target concentration ratio >1 (in the case of tazobactam and avibactam) or a free area under the concentration-to-time curve (*f*AUC)/MIC ratio >24 (in the case of vaborbactam) were achieved. The AUC of vaborbactam was calculated as AUC (mg·h/L) = dose (mg/24 h)/clearance (L/h), where clearance was equal to the infusion rate (mg/h)/Css (mg/L). The target concentration ratio corresponded to the fixed BLI target concentration defined by the EUCAST for testing the *in vitro* standard susceptibility of each BL/BLIc, namely, 4 mg/L for tazobactam or avibactam and 8 mg/L for vaborbactam.

The following demographic and clinical data were recorded for each patient: age, weight, sex, underlying onco-haematological disease, type and date of HSCT, type of chemotherapy regimens, type and site of infection, treatment length, previous antibiotic treatment, concomitant treatment with other antibiotics or antifungals. Clinical chemistry, pharmacological and microbiological data included serum creatinine (SCr), C-reactive protein (C-RP), ANC, beta-lactam Css, eventual bacterial clinical isolate with MIC, rectal swab colonization findings at admission. Estimated glomerular filtration rate was assessed by means of CKD-EPI formula.^30^ Patients having eGFR >120 (if female) or 130 (if male) mL/min/1.73 m^2^ were defined as having augmented renal clearance (ARC).^31^ Clinical outcome was recorded at the end of treatment.

### Assessment of outcomes

The primary study outcome was to assess the likelihood of aggressive PK/PD target attainment with the different beta-lactams or BL/BLIc. The secondary outcome was the overall clinical response at the end of treatment. Clinical cure was defined as the composite satisfaction of all the following criteria: defervescence lasting for at least 24 h, microbiological eradication documented by negative blood cultures (only in case of microbiological documented infections), resolution of signs and symptoms of infections and no need for escalating therapy.

### Ethics

This study was conducted in accordance with the Declaration of Helsinki. The Local Ethic Committee of the IRCCS, Azienda Ospedaliero-Universitaria di Bologna approved the protocol (no. 308/2021/Oss/AOUBo on 17 May 2021, EM232–2022_308/2021/Oss/AOUBo on 16 March 2022, EM449–2023_308/2021/Oss/AOUBo on 17 April 2024 and Ce AVEC: 272/2024/Sper/AOUBo on 16 May 2024). All patients provided written informed consent.

### Statistical analysis

The Kolmogorov–Smirnov test was used to assess normal or non-normal distribution of data. Accordingly, mean ± SD or median and IQR were used for the descriptive statistics. Differences in categorical variables were explored by Fisher’s exact test or *χ*^2^ test. Multivariate binary logistic regression analysis was performed to test the possible association between aggressive PK/PD target attainment and clinical variables pooled for all antibiotics with ORs and 95% CIs. Covariates with a *P* value of ≤0.20 at the univariate analysis were included in the multivariate analysis. All statistical analyses were performed using STATA (version 19).

## Results

**Figure 1.**
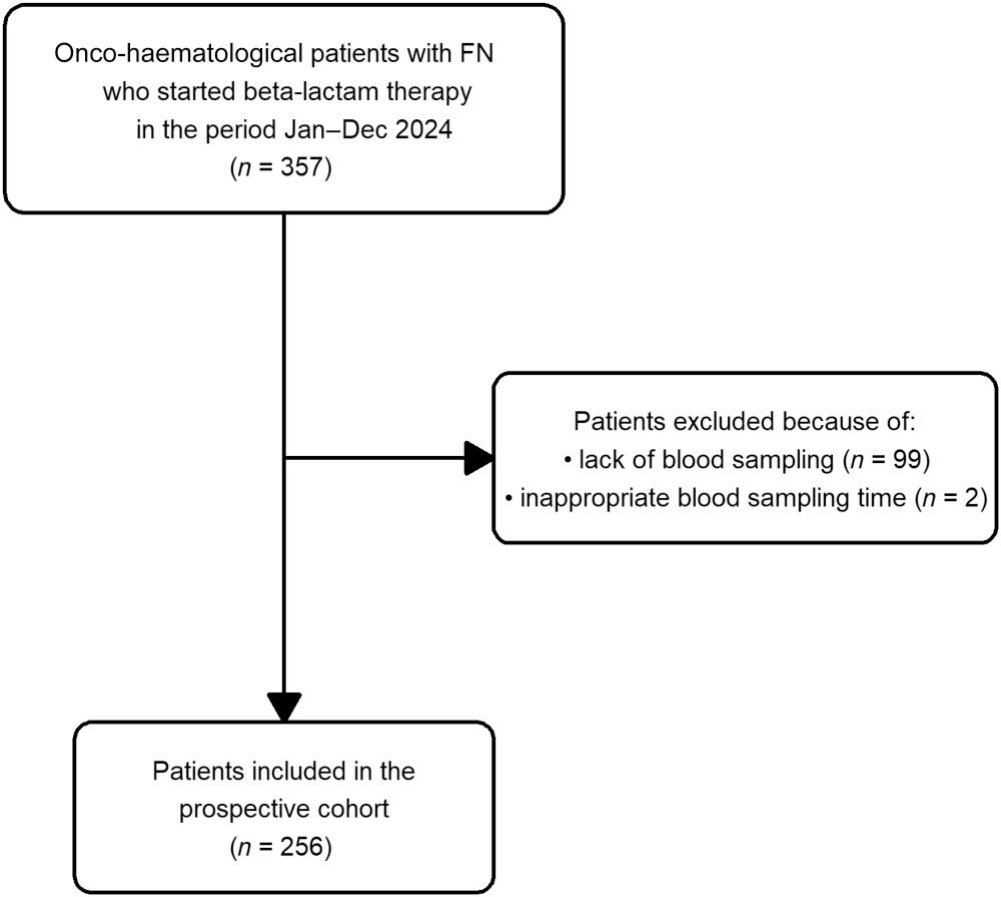
Flow chart of patient inclusion and exclusion criteria.

Among the 357 onco-haematological patients prospectively enrolled, unfortunately 101 had to be excluded because blood sampling for TDM was lacking or being collected outside of the pre-defined temporal window (Figure 1). Overall, a total of 256 patients was definitely included whose demographic and clinical characteristics are summarized in Table 1. A direct comparison of the baseline characteristics of patients having versus not having TDM assessment is provided in Table S1 (available as Supplementary data at *JAC* Online). The features of the two groups were similar, apart from the proportion of meropenem use (40.6% versus 26.7%, *P* = 0.019) and of piperacillin/tazobactam use (46.5% versus 60.3%, *P* = 0.015). Median (min–max) age was 58 (19–80) years, with a male preponderance (162/256, 63.3%). Acute myeloid leukaemia and non-Hodgkin lymphomas accounted for most of the underlying onco-haematological malignancies (183/256, 71.5%). Almost half patients underwent HSCT (120/256, 46.9%) and more than two-thirds had severe neutropenia at baseline (197/256, 76.9%). Most patients received antibiotic treatment with piperacillin/tazobactam or meropenem (223/256, 87.1%) and had beta-lactams as monotherapy (150/256, 58.6%). Co-treatment with antifungals was extensively applied (223/256, 87.1%). The median (min–max) duration of beta-lactam treatment was 7 (3–44) days. Site of infection was identified in more than half of cases (133/256, 51.9%), with bloodstream infections and pneumonia being the most represented types (109/133, 81.9%). A total of 82 patients had targeted treatment against Gram-negative documented infections (82/256, 32.1%), with 73 being monomicrobial and nine polymicrobial. Clinical cure was obtained in 71.1% of cases (182/256).

**Table 1.** Demographic and clinical characteristics (*n* = 256)

Clinical variable	
Age (years)	58.0 (50.8–67.0)
Sex (M/F)	162/94 (63.3/36.7)
Weight (kg)	72 (63–80)
BMI (kg/m^2^)	24.2 (21.9–26.3)
eGFR (mL/min/1.73 m^2^)	98.0 (81.0–112.0)
Chronic renal failure (<30 mL/min/1.73 m^2^)	6 (2.3)
ARC	23 (8.9)
Previous antibiotic in last 3 months	126 (49.2)
MDR rectal colonization at admission	95 (37.1)
ESBL-producing pathogens	65 (25.4)
KPC-producing pathogens	9 (3.5)
OXA48-producing pathogens	8 (3.1)
NDM-producing pathogens	1 (0.4)
VRE	12 (4.7)
Underlying haematological malignancy	
Acute myeloid leukaemia	102 (39.8)
Non-Hodgkin lymphoma	81 (31.7)
Multiple myeloma	25 (9.8)
Acute lymphoblastic leukaemia	27 (10.5)
Hodgkin lymphoma	10 (3.9)
Myelodysplastic syndrome	3 (1.2)
Others	8 (3.1)
Transplantation (*n* = 120)	
Allogenic transplantation	58 (22.7)
CAR-T	40 (15.7)
Autologous transplantation	22 (8.6)
Type of chemotherapy regimen	
Consolidation for acute leukaemia	86 (33.6)
Salvage chemotherapy	81 (31.6)
Induction for acute leukaemia	73 (28.5)
Other	16 (6.3)
Beta-lactam treatment	
Empirical	174 (67.9)
Targeted	82 (32.1)
Site of infection	
Unknown	123 (48.1)
Bloodstream infection	85 (33.2)
Community/hospital acquired pneumonia	24 (9.4)
Enterocolitis	10 (3.9)
Urinary tract infection	8 (3.1)
Acute bacterial skin and skin structure infection	6 (2.3)
ANC at baseline	
<100 (cells/µL)	159 (62.1)
100–500 (cells/µL)	38 (14.8)
500–1000 (cells/µL)	17 (6.6)
>1000 (cells/µL)	42 (16.3)
Type of beta-lactam	
Piperacillin/tazobactam	119 (46.5)
Meropenem	104 (40.6)
Ceftazidime/avibactam	17 (6.6)
Meropenem/vaborbactam	5 (1.9)
Ceftolozane/tazobactam	4 (1.6)
Cefiderocol	5 (1.9)
Ceftazidime	2 (0.8)
Duration of antibiotic treatment (days)	7.0 (5.0–9.0)
Combination therapy with	
Other anti-Gram-negative agents	32 (12.5)
Anti-Gram-positive agents	77 (30.1)
Antifungals	223 (87.1)

Continuous data are presented with median (IQR) and categorical variables as count (%).

**Figure 2.**
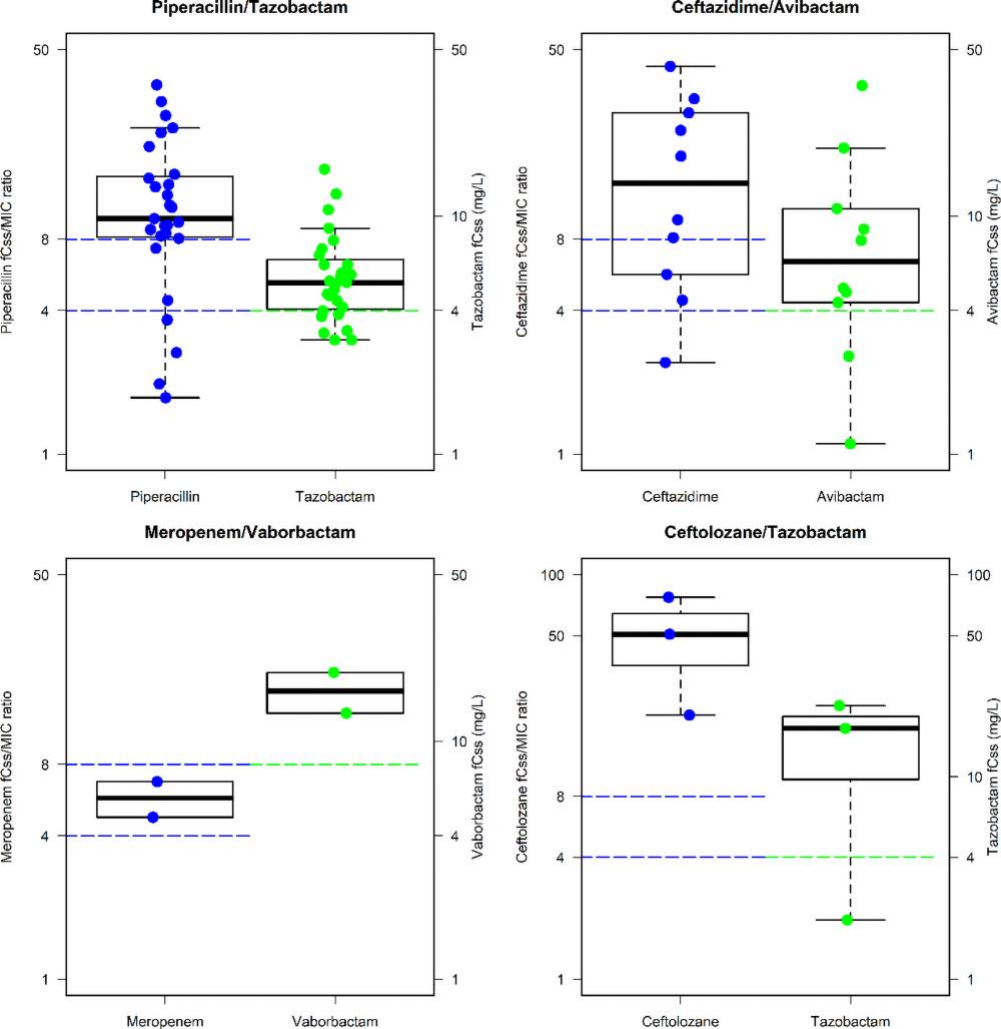
Box and whiskers plots of the *f*Css/MIC ratio of the beta-lactam and of the *f*Css of the coupled BLI for piperacillin/tazobactam, ceftazidime/avibactam, meropenem/vaborbactam and ceftolozane/tazobactam among patients receiving targeted therapy. The horizontal blue and green lines represent the threshold of beta-lactam *f*Css/MIC ratio and the BLI *f*Css, respectively.

Trends of aggressive PK/PD target attainment with the different CI beta-lactams in patients receiving targeted or empirical treatment are summarized in Tables 2 and 3, respectively. Among the 82 patients having targeted treatment, the aggressive PK/PD target was always attained with all the CI beta-lactams, except for piperacillin/tazobactam (62.9%) and ceftazidime/avibactam (80%). Non-attainment was always due to insufficient concentrations of the BLI in both BL/BLIc, namely tazobactam (*n* = 10) or avibactam (*n* = 3). The distributions of the PK/PD target attained by the different BL/BLIc in patients having targeted treatment is depicted in Figure 2.

**Table 2.** Distribution of the aggressive PK/PD target attainment with different continuous-infusion beta-lactams in patients receiving targeted therapy (*n* = 82)

Beta-lactam	Number of patients	Dose (g/daily)	eGFR (mL/min/1.73 m^2^)	Beta-lactam *f*Css (mg/L)	Beta-lactam inhibitor *f*Css (mg/L)	Aggressive PK/PD target attainment
PTZ	27	18 (18–18)	97 (35–129.3)	42.6 (34.4–58.8)	5.3 (4.1–6.5)	17/27 (62.9)
MEM	34	4 (0.5–6)	103.5 (24–221)	13.4 (9.5–19.7)	NA	34/34 (100)
CAZ/AVI	10	7 (3.75–10)	98.5 (29–107)	21.0 (16.6–21.4)	6.4 (4.4–10.3)	8/10 (80)
MEM/VAB	2	12 (12–12)	96.5 (75–118)	23.0 (21.1–25.0)	16.3 (14.7–17.9)	2/2 (100)
TOL/TAZ	3	9 (9–9)	82 (49–84)	38.7 (32.0–59.8)	17.4 (9.7–20.0)	3/3 (100)
CFD	5	6 (6–6)	95 (44–111)	24.7 (22.8–32.3)	NA	5/5 (100)
CAZ	1	4	85.7	18.9	NA	1/1 (100)

Continuous data are presented with median (min–max range) and categorical variables as count (%).

CAZ, ceftazidime; CAZ/AVI, ceftazidime/avibactam; CFD, cefiderocol; MEM, meropenem; MEM/VAB, meropenem/vaborbactam; PTZ, piperacillin/tazobactam.

**Table 3.** Distribution of aggressive PK/PD target attainment with different continuous-infusion beta-lactams in patients receiving empirical treatment (*n* = 174)

Beta-lactam	Number of patients	Dose (g/daily)	eGFR (mL/min/1.73 m^2^)	Beta-lactam *f*Css (mg/L)	Beta-lactam inhibitor *f*Css (mg/L)	Aggressive PK/PD target attainment
PTZ	92	18 (9–20.5)	95 (14–150)	36.6 (27.9–52.6)	4.9 (3.3–6.4)	49/92 (53.3)
MEM	70	4 (1–6)	102.5 (7–149)	11.5 (7.6–17.4)	NA	49/70 (70.0)
CAZ/AVI	7	7.5 (6–7.5)	102 (70–121)	16.2 (9.1–29.8)	3.3 (2.0–5.6)	1/7 (14.3)
MEM/VAB	3	12 (12–12)	115 (84–133)	8.1 (7.0–10.0)	7.2 (5.6–7.8)	0/3 (0)
TOL/TAZ	1	9	107	36.5	4.8	1/1 (100)
CAZ	1	6	101	9.9	NA	0/1 (0)

Continuous data are presented with median (min–max range) and categorical variables as count (%).

CAZ, ceftazidime; CAZ/AVI, ceftazidime/avibactam; CFD, cefiderocol; MEM, meropenem; MEM/VAB, meropenem/vaborbactam; PTZ, piperacillin/tazobactam.

No patient was empirically treated with cefiderocol.

**Figure 3.**
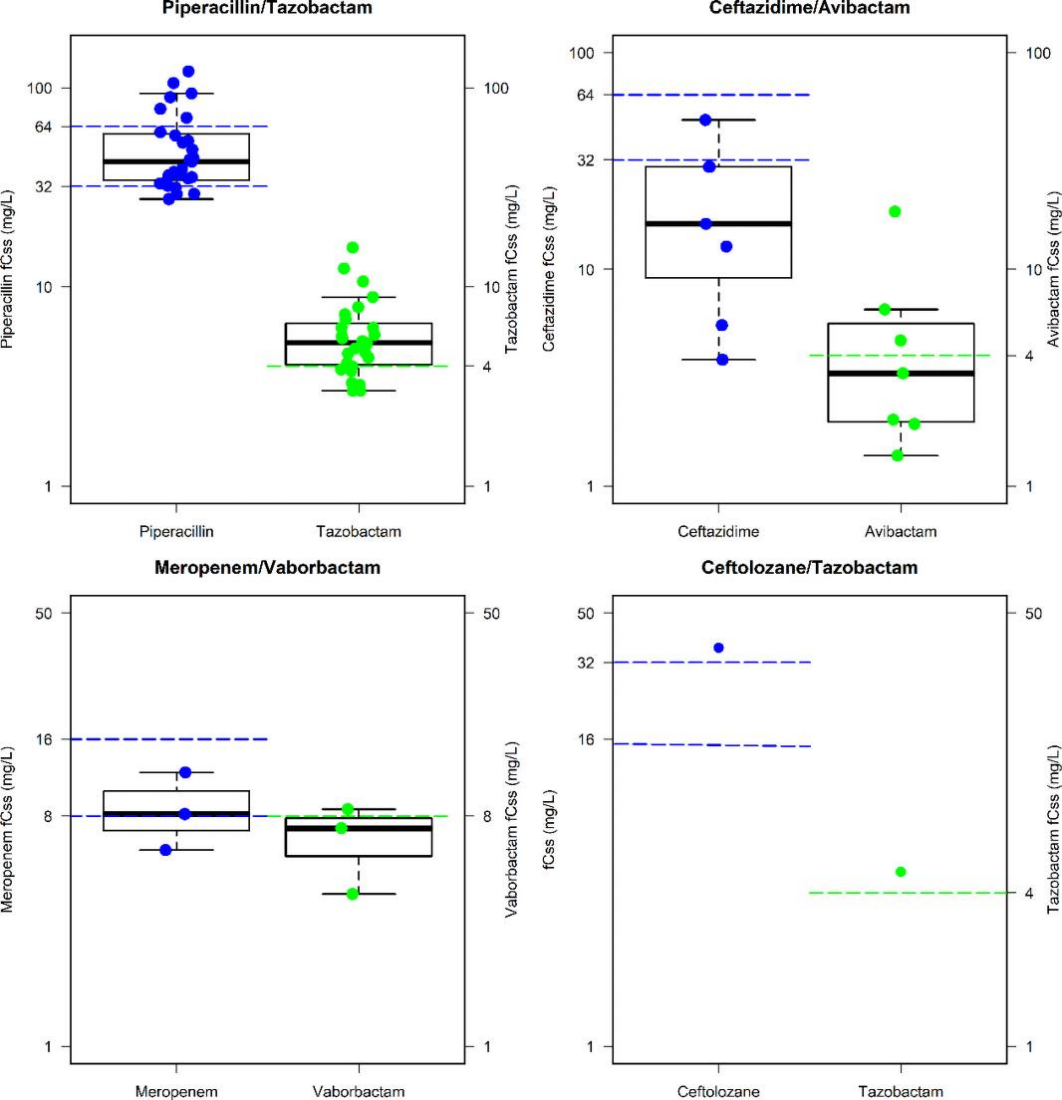
Box and whiskers plots of the *f*Css of the beta-lactam and of the coupled BLI for piperacillin/tazobactam, ceftazidime/avibactam, meropenem/vaborbactam and ceftolozane/tazobactam among patients receiving empirical therapy. The horizontal blue and green lines represent the threshold of beta-lactam *f*Css and the BLI *f*Css, respectively.

Among the 174 patients receiving empirical treatment, the aggressive PK/PD target was attained with all the other beta-lactams in only <70% of cases, except for ceftolozane/tazobactam (100%). Target non-attainment was due to insufficient concentrations of the beta-lactam for both meropenem alone (*n* = 21/70) and meropenem/vaborbactam (*n* = 3/3), and to insufficient concentrations of the BLI for piperacillin/tazobactam and for ceftazidime/avibactam, namely tazobactam (*n* = 43/92) and avibactam (*n* = 6/7), respectively. The distributions of the PK/PD target attained by the different BL/BLIc in patients having targeted treatment is depicted in Figure 3.

Multivariate logistic regression analysis testing potential predictors of aggressive PK/PD target attainment or non-attainment is reported in Table 4. ARC (OR 12.288 95%CI 3.314–45.569, *P* < 0.001), male sex(OR 3.792 95%CI 1.926–7.464, *P* < 0.001) and younger age (<65 yrs) (OR 2.402 95%CI 1.118–5161, *P* = 0.025) were risk factors associated with target non-attainment, whereas targeted therapy (OR 0.238 95%CI 0.112–0.505, *P* < 0.001) and treatment with meropenem (OR 0.204 95%CI 0.099–0.419, *P* < 0.001) were associated with higher probabilities of aggressive PK/PD target attainment.

**Table 4.** Univariate and multivariate logistic regression analysis comparing aggressive PK/PD target attainment with non-attainment with continuous-infusion beta-lactams in onco-haematological patients (*n* = 256)

Variables	PK/PD target attainment (*n *= 169)	PK/PD target non-attainment (*n* = 87)	Univariate *P* value	Multivariate analysis
Age <65 yrs	111 (65.7)	75 (86.2)	0.001	OR 2.402 (1.118–5.161), *P* = 0.025
Sex (male)	95 (56.2)	67 (77.0)	0.001	OR 3.792 (1.926–7.464), *P* < 0.001
BMI >30	20 (11.8)	11 (12.6)	0.851	
ARC	6 (3.6)	17 (19.5)	<0.001	OR 12.288 (3.314–45.569), *P* < 0.001
Acute leukaemia	82 (48.5)	46 (52.9)	0.510	
HSCT	71 (42.0)	49 (56.3)	0.031^a^	
Targeted therapy	69 (40.8)	13 (14.9)	<0.001	OR 0.238 (0.112–0.505), *P* < 0.001
ANC <1000 cells/µL	135 (79.9)	80 (91.9)	<0.001^a^	
Treatment with piperacillin/tazobactam	66 (39.1)	53 (60.9)	0.234	
Treatment with meropenem	83 (49.1)	21 (24.1)	<0.001	OR 0.204 (0.099–0.419), *P* < 0.001
Treatment with ceftazidime/avibactam	9 (5.3)	8 (9.2)	0.808	

ANC, absolute neutrophil count; BMI, body mass index.

^a^HSCT and ANC <1000 cells/µL were significant at univariate analysis but not after multivariate regression analysis.

Aggressive PK/PD target attainment was not associated with significantly improved clinical outcome at the univariate analysis either in the case of empirical treatment [73.4% (94/128) versus 71.7% (33/46), *P* = 0.847] or in that of targeted treatment [67.5% (52/77) versus 60.0% (3/5), *P* = 1.000].

## Discussion

This study first assessed the likelihood of aggressive PK/PD target attainment among onco-haematological patients having FN treated with different beta-lactams administered by CI. Overall, the findings showed that when delivering standard dosing regimens of beta-lactams by CI an aggressive PK/PD target attainment was very likely with most of them when dealing with targeted treatment, and much less likely when dealing with empirical treatment. Noteworthy, piperacillin/tazobactam, namely the most widely used agent, was the only one with more than one-third of patients showing non-attainment in both settings (namely 37.1% and 46.7%, respectively). Moreover, the results showed the worrisome risk of BLI underexposure, which may compromise the clinical efficacy of BL/BLIc treatment.

A crucial advantage of targeting aggressive PK/PD target for beta-lactams may be that of a possible prevention of resistance development. This was just shown to be helpful among the critically ill patients having documented Gram-negative infections. A recent meta-analysis showed that attaining an aggressive PK/PD target was very protective against the risk of beta-lactam resistance development compared with a conservative PK/PD target (OR 0.06 95%CI 0.01–0.29).^14^ Regarding the setting of onco-haematological patients, it should not be overlooked that this could be a very relevant issue considering that recent beta-lactam use within 3 months was shown to be a major risk factor of colonization by either ESBL-producing-^32^ or carbapenem-resistant-*Enterobacterales* (CRE),^33,34^ the prevalence of the latter possibly being as high as 11.4%.^34^ Notably, a prospective, monocentric, interventional study showed that targeting meropenem exposure to an aggressive PK/PD target in onco-haematological patients with FN was shown to be an effective approach for minimizing the emergence of CRE.^35^ Interestingly, it was shown that none of the 63 out of 75 patients (84%) who were readmitted in hospital during a 3 month follow-up period after receiving real-time TDM-guided CI meropenem for treating a FN episode had colonization by CRE at rectal swabs.^35^ This is particularly relevant, as meropenem use was primarily associated to selection of carbapenem resistance,^36^ as well as to increased GVHD-related mortality in onco-haematological patients undergoing HSCT due to its strong anaerobic activity.^37^

Unfortunately, the TDM-guided approach of beta-lactams nowadays is still limited to some centres and not yet widely implemented. Consequently, knowing the likelihood of aggressive PK/PD target attainment during the first week of treatment with CI of standard dosing regimens of different beta-lactams may be a helpful tool for clinicians in estimating the possibility of minimizing resistance development in clinical practice. The findings showed that the probability was in general high when dealing with targeted treatment. However, the risk of non-attainment may be especially likely irrespective of CI administration among younger onco-haematological male patients having ARC. ARC is a prevalent condition among FN patients, ranging 16.4%–26.6%.^38,39^ Indeed, the prevalence of ARC detected in our cohort was lower than this (8.9%), but the true prevalence might have been biased by the fact that the creatinine clearance was estimated by means of a formula rather than measured. In any case, this is a worrying condition during beta-lactam treatment, since it may expedite the renal clearance of these hydrophilic agents. Consequently, intensifying the CI dosing regimens of the beta-lactams could be an effective solution for attaining aggressive PK/PD targets even in FN patients having ARC. In this regard, we showed previously by means of a population pharmacokinetic study that delivering an intensified meropenem dose of 1.25 g q6h by CI may be an effective strategy for attaining an aggressive PK/PD target in most FN patients with ARC having infections caused by susceptible pathogens.^40^ Conversely, intensifying the CI dosing regimens of piperacillin/tazobactam and ceftazidime/avibactam could not always suffice for attaining an aggressive PK/PD target in FN patients with ARC. Lacking specific data in the context of FN patients on this issue, two Monte Carlo simulation studies carried out among critically ill patients may be helpful.^41^ One showed that even delivering a piperacillin/tazobactam dose as high as 22.5 g/daily by CI was ineffective in attaining an aggressive PK/PD target among ARC critically ill patients having infections caused by susceptible pathogens.^41^ Conversely, the other showed that delivering an intensified ceftazidime/avibactam dose of 10 g/daily by CI was predicted to grant a high probability (>80%) of aggressive PK/PD target attainment among ARC critically ill patient having KPC-KP- or OXA-48-producing *Enterobaterales* related infections.^42^ Lacking evidence on specific dosing regimens to be recommended for BL/BLIc in FN onco-haematological patients, clinicians should rely on TDM whenever feasible for minimizing the risk of aggressive PK/PD target non-attainment.

Less agreement exists about the role that an aggressive PK/PD target attainment may have in maximizing the clinical efficacy of beta-lactam treatment in FN patients. Data are limited and available evidence is only indirect, by coming mainly from studies assessing the impact that may have an extended infusion (EI) delivery of standard dosing regimens of beta-lactams. A single-centre randomized clinical trial was designed to compare the impact of EI with intermittent infusion (II) on the efficacy of piperacillin/tazobactam and ceftazidime in the treatment of high-risk FN patients.^43^ At the intention-to-treat analysis among 105 included patients the overall response rate was significantly higher with EI than with II (74.4% versus 55.1%; *P* = 0.044), and in documented infections compared with empirical treatment (68.4% versus 35.7%; *P* = 0.039).^43^ A retrospective observational study carried out among 164 patients having FN treated with meropenem showed that EI led to better clinical outcome than conventional II and was independently associated with clinical success at day 5 (OR 3.13. 95%CI 1.61–6.10), faster defervescence, more rapid decrease of inflammatory biomarkers and less need for additional antibiotics.^44^ Similarly, among 193 patients with haematological malignancies having FN treated with cefepime, EI was associated with a more rapid defervescence compared with standard II (48 h versus 70 h, *P* = 0.005).^45^ Conversely, a randomized, multicentre, open-label, superiority clinical trial showed that the success rate at day 5 of meropenem in the empirical treatment of FN patients did not differ in those receiving EI versus II (risk difference, −12.4%; 95% CI, −29.4 to 4.7; *P* 0.17).^46^

Indeed, these conflicting findings highlight how difficult may be assessing the clinical outcome of beta-lactam treatment in FN patients. It should not be overlooked that treatment success may be biased by several co-treatments in this population, namely with anti-Gram-positives and/or with antifungals, which may mask the beneficial effect of an aggressive PK/PD target attainment against Gram-negatives.

We acknowledge some limitations of this study. The heterogenicity of the patients’ clinical conditions prevented us from finding a clear association between aggressive PK/PD target attainment with beta-lactams and clinical outcome of FN. Aggressive PK/PD target attainment is a goal widely adopted nowadays among critically ill patients, but needs more confirmatory findings among FN onco-haematological patients, as it may not uniformly translate into improved clinical outcomes across all pathogens and drug combinations. Specifically, when dealing with empirical treatment, using clinical breakpoints as the referral MIC value does not necessarily correspond to the effective PK/PD target. Assessing a single TDM did not allow us to capture intra-patient variability related to changing clinical conditions and renal function over time. Sampling within a pre-defined timeframe may have introduced a bias, as patients discontinuing therapy early or deteriorating clinically may have been underrepresented. The findings of the regression analysis may be biased by the high proportion of patients excluded because not undergoing TDM. The higher proportion of PK/PD target attainment among targeted therapy may be explained by the fact that the documented MIC values of the clinical isolates were frequently much lower than the respective clinical breakpoints. The effect of aggressive PK/PD target attainment on preventing rectal colonization at follow-up by MDR pathogens was not assessed. Only total beta-lactam concentrations were measured and this might have affected proper estimation of the free fractions. Conversely, the prospective study design assessing the PK/PD target attainment of different beta-lactams both in the empiric and in the targeted treatment of FN onco-haematological patients is an innovative strength of this work.

In conclusion, our findings showed that delivering the standard dosing regimens of different beta-lactams by CI may help attaining an aggressive PK/PD target in FN patients having targeted treatment. Young male patients with ARC, being at higher risk of non-attainment, should benefit from strategies focused on more intensified dosing regimens. Since this study was mainly aimed at assessing the likelihood of PK/PD target attainment of CI beta-lactams under standard dosing regimens, it was underpowered for testing its impact in improving clinical efficacy. Accordingly, a prospective study is currently in the planning stage for assessing the real impact that an aggressive PK/PD target attainment of beta-lactams might have on clinical outcome and in preventing the rectal colonization by MDR pathogens producing resistance to the used beta-lactam in this setting.

## Supplementary Material

dkag183_Supplementary_Data

## Transparency declarations

P.G.C. has received grant from Angelini and Shionogi. P.V. has served as a consultant for Advanz Pharma, Angelini, bioMérieux, Gilead, Merck Sharp & Dohme, InfectoPharm, Pfizer, Shionogi, Thermo-Fisher, Venatorx and Viatris, and served on the speaker’s bureau for Advanz Pharma, Angelini, Gilead, Merck Sharp & Dohme, InfectoPharm, Menarini, Pfizer, Shionogi and Viatris outside the submitted work. F.P. has served as a consultant for Advanz Pharma, Angelini, Gilead, Merck Sharp & Dohme, Pfizer and Viatris outside the submitted work, and participated in the speaker’s bureau for Angelini, Gilead, Menarini, Merck Sharp & Dohme, InfectoPharm, Pfizer, Shionogi and Viatris. The other authors have no conflicts of interest to declare.

## Supplementary data

Table S1 is available as Supplementary data at *JAC* Online.

## References

[1] Holmes CL, Albin OR, Mobley HLT et al Bloodstream infections: mechanisms of pathogenesis and opportunities for intervention. Nat Rev Microbiol 2025; 23: 210–24. 10.1038/s41579-024-01105-2 39420097 PMC12519459

[2] Er AG, Aslan AT, Mikulska M et al Prevention and treatment of bacterial infections in patients with haematological cancers and haematopoietic stem cell transplantation: headways and shortcomings. Clin Microbiol Infect 2025; 31: 24–8. 10.1016/j.cmi.2024.09.015 39332598

[3] Sandherr M, Stemler J, Schalk E et al 2024 update of the AGIHO guideline on diagnosis and empirical treatment of fever of unknown origin (FUO) in adult neutropenic patients with solid tumours and hematological malignancies. Lancet Reg Health Eur 2025; 51: 101214. 10.1016/j.lanepe.2025.101214 39973942 PMC11836497

[4] Kochanek M, Schalk E, von Bergwelt-Baildon M et al Management of sepsis in neutropenic cancer patients: 2018 guidelines from the infectious diseases working party (AGIHO) and intensive care working party (iCHOP) of the German Society of Hematology and Medical Oncology (DGHO). Ann Hematol 2019; 98: 1051–69. 10.1007/s00277-019-03622-0 30796468 PMC6469653

[5] Schmidt-Hieber M, Teschner D, Maschmeyer G et al Management of febrile neutropenia in the perspective of antimicrobial de-escalation and discontinuation. Expert Rev Anti Infect Ther 2019; 17: 983–95. 10.1080/14787210.2019.1573670 30686067

[6] de la Court JR, Bruns AHW, Roukens AHE et al The Dutch working party on antibiotic policy (SWAB) recommendations for the diagnosis and management of febrile neutropenia in patients with cancer. Infect Dis Ther 2022; 11: 2063–98. 10.1007/s40121-022-00700-1 36229765 PMC9669256

[7] Averbuch D, Vanbiervliet Y, Baccelli F et al Empirical and targeted antimicrobial therapy in patients with febrile neutropenia and haematological malignancy or after haematopoietic cell transplantation: recommendations from the 10th European Conference on Infections in Leukaemia. Lancet Infect Dis 2025: S1473-3099(25)00619-X. 10.1016/S1473-3099(25)00619-X. Epub ahead of print. 41314221

[8] Trecarichi EM, Giuliano G, Cattaneo C et al Bloodstream infections caused by Escherichia coli in onco-haematological patients: risk factors and mortality in an Italian prospective survey. PLoS One 2019; 14:e0224465. 10.1371/journal.pone.0224465 31661507 PMC6818756

[9] Correa AF, Guasti P, Fuenmayor-González L et al Microbiological characterization of bacteremia in patients with chemotherapy-induced febrile neutropenia: systematic review and meta-analysis. Ther Adv Infect Dis 2025; 12:20499361251376123. 10.1177/20499361251376123 41084620 PMC12515338

[10] Pagano L, Caira M, Trecarichi EM et al Carbapenemase-producing *Klebsiella pneumoniae* and hematologic malignancies. Emerg Infect Dis 2014; 20: 1235–6. 10.3201/eid2007.130094 24960464 PMC4073839

[11] Trecarichi EM, Pagano L, Candoni A et al Current epidemiology and antimicrobial resistance data for bacterial bloodstream infections in patients with hematologic malignancies: an Italian multicentre prospective survey. Clin Microbiol Infect 2015; 21: 337–43. 10.1016/j.cmi.2014.11.022 25595706

[12] Craig WA . Pharmacokinetic/pharmacodynamic parameters: rationale for antibacterial dosing of mice and men. Clin Infect Dis 1998; 26: 1–10; quiz 1-2. 10.1086/516284 9455502

[13] Cojutti PG, Gatti M, Rinaldi M et al Impact of maximizing Css/MIC ratio on efficacy of continuous infusion meropenem against documented Gram-negative infections in critically ill patients and population pharmacokinetic/pharmacodynamic analysis to support treatment optimization. Front Pharmacol 2021; 12:781892. 10.3389/fphar.2021.781892 34955851 PMC8694396

[14] Gatti M, Cojutti PG, Pea F. Impact of attaining aggressive vs. conservative PK/PD target on the clinical efficacy of beta-lactams for the treatment of gram-negative infections in the critically ill patients: a systematic review and meta-analysis. Crit Care 2024; 28: 123. 10.1186/s13054-024-04911-5 38627763 PMC11020314

[15] Gatti M, Cojutti PG, Pascale R et al Assessment of a PK/PD target of continuous infusion beta-lactams useful for preventing microbiological failure and/or resistance development in critically ill patients affected by documented Gram-negative infections. Antibiotics 2021; 10: 1311. 10.3390/antibiotics10111311 34827249 PMC8615220

[16] Dulhunty JM, Brett SJ, De Waele JJ et al Continuous vs intermittent β-lactam antibiotic infusions in critically ill patients with sepsis: the BLING III randomized clinical trial. JAMA 2024; 332: 629–37. 10.1001/jama.2024.9779 38864155 PMC11170452

[17] Gatti M, Rinaldi M, Cojutti PG et al A pre–post quasi-experimental study of antimicrobial stewardship exploring the impact of a multidisciplinary approach aimed at attaining an aggressive joint pharmacokinetic/pharmacodynamic target with ceftazidime/avibactam on treatment outcome of KPC-producing *Klebsiella pneumoniae* infections and on ceftazidime/avibactam resistance development. Antimicrob Agents Chemother 2025; 69:e0048825. 10.1128/aac.00488-25 40476843 PMC12217479

[18] Stohs EJ, Abbas A, Freifeld A. Approach to febrile neutropenia in patients undergoing treatments for hematologic malignancies. Transpl Infect Dis 2024; 26:e14236. 10.1111/tid.14236 38349035

[19] Ramirez S, Scapaticci M, Barbella F et al Development of a rapid LC-MS/MS method for simultaneous quantification of ten commonly used antibiotic drugs in human serum. J Pharm Biomed Anal 2024; 244:116119. 10.1016/j.jpba.2024.116119 38579409

[20] Sillén H, Mitchell R, Sleigh R et al Determination of avibactam and ceftazidime in human plasma samples by LC-MS. Bioanalysis 2015; 7: 1423–34. 10.4155/bio.15.76 26168250

[21] Conti M, Giorgi B, Gatti M et al Development and validation of a sensitive liquid chromatography-tandem mass spectrometry method for therapeutic drug monitoring of ceftolozane and tazobactam in human plasma microsamples. Ther Drug Monit 2024; 46: 756–63. 10.1097/FTD.0000000000001236 39115870

[22] Barone R, Conti M, Giorgi B et al Fast and sensitive method for simultaneous quantification of meropenem and vaborbactam in human plasma microsamples by liquid chromatography-tandem mass spectrometry for therapeutic drug monitoring. Antibiotics 2023; 12: 719. 10.3390/antibiotics12040719 37107082 PMC10135283

[23] Barone R, Conti M, Cojutti PG et al Fast and sensitive analysis of cefiderocol in human plasma microsamples by liquid chromatography-isotope dilution tandem mass spectrometry for therapeutic drug monitoring. Antibiotics 2023; 12: 213. 10.3390/antibiotics12020213 36830124 PMC9952754

[24] Wong G, Briscoe S, Adnan S et al Protein binding of β-lactam antibiotics in critically ill patients: can we successfully predict unbound concentrations? Antimicrob Agents Chemother 2013; 57: 6165–70. 10.1128/AAC.00951-13 24080664 PMC3837907

[25] Sy SKB, Zhuang L, Sy S et al Clinical pharmacokinetics and pharmacodynamics of ceftazidime-avibactam combination: a model-informed strategy for its clinical development. Clin Pharmacokinet 2019; 58: 545–64. 10.1007/s40262-018-0705-y 30097887

[26] Cho JC, Fiorenza MA, Estrada SJ. Ceftolozane/tazobactam: a novel cephalosporin/β-lactamase inhibitor combination. Pharmacotherapy 2015; 35: 701–15. 10.1002/phar.1609 26133315

[27] Katsube T, Echols R, Wajima T. Pharmacokinetic and pharmacodynamic profiles of cefiderocol, a novel siderophore cephalosporin. Clin Infect Dis 2019; 69: S552–8. 10.1093/cid/ciz828 31724042 PMC6853762

[28] Griffith DC, Sabet M, Tarazi Z et al Pharmacokinetics/pharmacodynamics of vaborbactam, a novel beta-lactamase inhibitor, in combination with meropenem. Antimicrob Agents Chemother 2019; 63:e01659-18. 10.1128/AAC.01659-18 30397063 PMC6325214

[29] Gatti M, Rinaldi M, Laici C et al Impact of attaining an aggressive pharmacokinetic-pharmacodynamic target on the clinical efficacy of continuous infusion β-lactam therapy for early posttransplant Gram-negative infections in critically ill orthotopic liver transplant recipients: an interim analysis of a 3-year prospective, observational study. J Infect Dis 2025; 232: e875–85. 10.1093/infdis/jiaf048 39854631 PMC12718055

[30] Levey AS, Stevens LA, Schmid CH et al A new equation to estimate glomerular filtration rate. Ann Intern Med 2009; 150: 604–12. 10.7326/0003-4819-150-9-200905050-00006 19414839 PMC2763564

[31] Ye X, Yuan Q, Du Z et al Prevalence and risk factors for augmented renal clearance in neurocritical ill patients. J Clin Lab Anal 2025; 39:e70047. 10.1002/jcla.70047 40394958 PMC12144577

[32] Kömürcü B, Tükenmez Tigen E, Toptaş T et al Rectal colonization with multidrug-resistant gram-negative bacteria in patients with hematological malignancies: a prospective study. Expert Rev Hematol 2020; 13: 923–7. 10.1080/17474086.2020.1787145 32574123

[33] Chiotos K, Tamma PD, Flett KB et al Multicenter study of the risk factors for colonization or infection with carbapenem-resistant Enterobacteriaceae in children. Antimicrob Agents Chemother 2017; 61:e01440-17. 10.1128/AAC.01440-17 28971864 PMC5700345

[34] Demiraslan H, Cevahir F, Berk E et al Is surveillance for colonization of carbapenem-resistant gram-negative bacteria important in adult bone marrow transplantation units? Am J Infect Control 2017; 45: 735–9. 10.1016/j.ajic.2017.01.006 28214159

[35] Cojutti PG, Lazzarotto D, Candoni A et al Real-time TDM-based optimization of continuous-infusion meropenem for improving treatment outcome of febrile neutropenia in oncohaematological patients: results from a prospective, monocentric, interventional study. J Antimicrob Chemother 2020; 75: 3029–37. 10.1093/jac/dkaa267 32681168 PMC7678894

[36] Moghnieh R, Abdallah D, Jadayel M et al Epidemiology, risk factors, and prediction score of carbapenem resistance among inpatients colonized or infected with 3rd generation cephalosporin resistant Enterobacterales. Sci Rep 2021; 11: 14757. 10.1038/s41598-021-94295-1 34285312 PMC8292374

[37] Ito H, Okamura Y, Tomura Y et al Effect of antibiotics with anaerobic coverage on graft-versus-host disease in patients undergoing allogeneic hematopoietic stem cell transplantation: a systematic review and meta-analysis. Transpl Infect Dis 2025; 27:e70049. 10.1111/tid.70049 40298411 PMC12416444

[38] Kondo N, Tanaka R, Tatsuta R et al Effect of augmented renal clearance and febrile neutropenia on initial trough level and clearance of teicoplanin. Ther Drug Monit 2025; 47: 385–92. 10.1097/FTD.0000000000001320 40145829

[39] Hirai K, Ishii H, Shimoshikiryo T et al Augmented renal clearance in patients with febrile neutropenia is associated with increased risk for subtherapeutic concentrations of vancomycin. Ther Drug Monit 2016; 38: 706–10. 10.1097/FTD.0000000000000346 27681114

[40] Cojutti PG, Candoni A, Lazzarotto D et al Population pharmacokinetics of continuous-infusion meropenem in febrile neutropenic patients with hematologic malignancies: dosing strategies for optimizing empirical treatment against *Enterobacterales* and *P. aeruginosa*. Pharmaceutics 2020; 12: 785. 10.3390/pharmaceutics12090785 32825109 PMC7560225

[41] Cojutti PG, Pai MP, Tonetti T et al Balancing the scales: achieving the optimal beta-lactam to beta-lactamase inhibitor ratio with continuous infusion piperacillin/tazobactam against extended spectrum beta-lactamase producing *Enterobacterales*. Antimicrob Agents Chemother 2024; 68:e0140423. 10.1128/aac.01404-23 38411995 PMC10994818

[42] Cojutti PG, Pai MP, Gatti M et al An innovative population pharmacokinetic/pharmacodynamic strategy for attaining aggressive joint PK/PD target of continuous infusion ceftazidime/avibactam against KPC- and OXA-48-producing *Enterobacterales* and preventing resistance development in critically ill patients. J Antimicrob Chemother 2024; 79: 2801–8. 10.1093/jac/dkae290 39159014

[43] Ram R, Halavy Y, Amit O et al Extended vs bolus infusion of broad-spectrum β-lactams for febrile neutropenia: an unblinded, randomized trial. Clin Infect Dis 2018; 67: 1153–60. 10.1093/cid/ciy258 29608680

[44] Fehér C, Rovira M, Soriano A et al Effect of meropenem administration in extended infusion on the clinical outcome of febrile neutropenia: a retrospective observational study. J Antimicrob Chemother 2014; 69: 2556–62. 10.1093/jac/dku150 24855125

[45] Crawford R, Perkins NB, Hobbs DA et al Time to defervescence evaluation for extended- vs. standard-infusion cefepime in patients with acute leukemia and febrile neutropenia. Pharmacotherapy 2022; 42: 798–805. 10.1002/phar.2728 36106434

[46] Laporte-Amargos J, Carmona-Torre F, Huguet M et al Efficacy of extended infusion of β-lactam antibiotics for the treatment of febrile neutropenia in haematologic patients (BEATLE): a randomized, multicentre, open-label, superiority clinical trial. Clin Microbiol Infect 2025; 31: 211–9. 10.1016/j.cmi.2024.10.006 39433124

