# Supplementary material for: Likelihood of aggressive PK/PD target attainment of continuous-infusion beta-lactams during the first week of treatment of febrile neutropenia: findings from a 1-year prospective, monocentric study in onco-haematological patients
Source: J Antimicrob Chemother. 2026 Jun 3;81(7):dkag183. doi: 10.1093/jac/dkag183 (PMC13232040; doi:10.1093/jac/dkag183)
Supplement: dkag183_Supplementary_Data [file dkag183_supplementary_data.docx]

**Supplemetary material**

**Likelihood of aggressive PK/PD target attainment of continuous infusion beta-lactams during the first week of treatment of febrile neutropenia: findings from a 1-year prospective, monocentric study in onco-hematologic patients**

| **Table S1.** Demographic and clinical characteristics of oncohematological patients with febrile neutropenia who started beta-lactam therapy in the period Jan-Dec 2024 (n=357) | | | | | | | | | |
| --- | --- | --- | --- | --- | --- | --- | --- | --- | --- |
| Clinical variable | | | | | | | Patients with TDM assessment (n=256) | Patients without TDM assessment (n=101) | p-value |
| Age (yrs) | | | | | | | 58.0 (50.8 – 67.0) | 58.0 (48.0 – 65.0) | 0.371 |
| Gender (M/F) | | | | | | | 162/94 (63.3/36.7) | 71/30 (70.3/29.7) | 0.219 |
| Weight (kg) | | | | | | | 72 (63 – 80) | 73 (62 – 82) | 0.499 |
| BMI (kg/m^2^) | | | | | | | 24.2 (21.9 – 26.3) | 24.5 (20.8 – 26.6) | 0.497 |
| eGFR (mL/min/1.73 m^2^) | | | | | | | 98.0 (81.0 – 112.0) | 97.5 (81.2 – 113.0) | 0.533 |
| Chronic renal failure (<30 mL/min/1.73 m^2^) | | | | | | | 6 (2.3) | 2 (1.9) | 1.000 |
| Augmented renal clearance (ARC) | | | | | | | 23 (8.9) | 9 (8.9) | 1.000 |
| Previous antibiotic in last 3 months | | | | | | | 126 (49.2) | 43 (42.6) | 0.290 |
| MDR rectal colonization at admission | | | | | | | 95 (37.1) | 30 (29.7) | 0.218 |
|  | ESBL-producing pathogens | | | | | | 65 (25.4) | 18 (17.8) | 0.164 |
|  | KPC-producing pathogens | | | | | | 9 (3.5) | 3 (2.9) | 1.000 |
|  | OXA48-producing pathogens | | | | | | 8 (3.1) | 2 (1.9) | 0.731 |
|  | NDM-producing pathogens | | | | | | 1 (0.4) | 3 (2.9) | 0.070 |
|  | VRE | | | | | | 12 (4.7) | 4 (3.9) | 1.000 |
| Underlying hematological malignancy | | | | | | |  |  |  |
|  | | Acute myeloid leukemia | | | | | 102 (39.8) | 34 (33.7) | 0.333 |
|  | | Non-Hodgkin lymphoma | | | | | 81 (31.7) | 42 (41.5) | 0.084 |
|  | | Multiple myeloma | | | | | 25 (9.8) | 9 (8.9) | 1.000 |
|  | | Acute Lymphoblastic leukemia | | | | | 27 (10.5) | 7 (6.9) | 0.423 |
|  | | Hodgkin lymphoma | | | | | 10 (3.9) | 6 (5.9) | 0.403 |
|  | | Myelodysplastic syndrome | | | | | 3 (1.2) | 1 (0.9) | 1.000 |
|  | | Others | | | | | 8 (3.1) | 2 (1.9) | 0.731 |
| Transplantation (n=120) | | | | | | |  |  |  |
|  | | | Allogenic transplantation | | | | 58 (22.7) | 14 (13.9) | 0.078 |
|  | | | CAR-T | | | | 40 (15.7) | 12 (11.9) | 0.409 |
|  | | | Autologous transplantation | | | | 22 (8.6) | 11 (10.9) | 0.544 |
| Type of chemotherapy regimen | | | | | | |  |  |  |
|  | | | Consolidation for acute leukemia | | | | 86 (33.6) | 27 (26.7) | 0.256 |
|  | | | Salvage chemotherapy | | | | 81 (31.6) | 35 (34.7) | 0.617 |
|  | | | Induction for acute leukemia | | | | 73 (28.5) | 29 (28.7) | 1.000 |
|  | | | Other | | | | 16 (6.3) | 10 (9.9) | 0.259 |
| Beta-lactam treatment | | | | | | |  |  |  |
|  | | | | | | Empirical | 174 (67.9) | 70 (69.3) | 0.899 |
|  | | | | | | Targeted | 82 (32.1) | 31 (30.7) | 0.899 |
| Site of infection | | | | | | |  |  |  |
|  | | | | | | Unknown | 123 (48.1) | 54 (53.5) | 0.411 |
|  | | | | | | Bloodstream infection | 85 (33.2) | 24 (23.8) | 0.097 |
|  | | | | | | Community/hospital acquired pneumonia | 24 (9.4) | 9 (8.9) | 1.000 |
|  | | | | | | Enterocolitis | 10 (3.9) | 8 (7.9) | 0.176 |
|  | | | | | | Urinary tract infection | 8 (3.1) | 3 (2.9) | 1.000 |
|  | | | | | | Acute bacterial skin and skin structure infection | 6 (2.3) | 3 (2.9) | 0.717 |
| ANC at baseline | | | | | | |  |  |  |
|  | | | | | | <100 (cells/µL) | 159 (62.1) | 53 (52.5) | 0.119 |
|  | | | | | | 100-500 (cells/µL) | 38 (14.8) | 18 (17.8) | 0.519 |
|  | | | | | | 500-1000 (cells/µL) | 17 (6.6) | 13 (12.9) | 0.087 |
|  | | | | | | >1000 (cells/µL) | 42 (16.3) | 17 (16.8) | 1.000 |
| Type of beta-lactam | | | | | | |  |  |  |
|  | | | | | Piperacillin/tazobactam | | 119 (46.5) | 61 (60.3) | 0.019 |
|  | | | | | Meropenem | | 104 (40.6) | 27 (26.7) | 0.015 |
|  | | | | | Ceftazidime/avibactam | | 17 (6.6) | 6 (5.9) | 1.000 |
|  | | | | | Meropenem/vaborbactam | | 5 (1.9) | 4 (3.9) | 0.154 |
|  | | | | | Ceftolozane/tazobactam | | 4 (1.6) | 0 (0) | 0.581 |
|  | | | | | Cefiderocol | | 5 (1.9) | 1 (0.9) | 1.000 |
|  | | | | | Ceftazidime | | 2 (0.8) | 2 (1.9) | 0.318 |
| Duration of antibiotic treatment (days) | | | | | | | 7.0 (5.0 – 9.0) | 5.0 (3.0 – 6.0) | 0.092 |
| Combination therapy with | | | | | | |  |  |  |
|  | | | | Other anti Gram-negative agents | | | 32 (12.5) | 20 (19.8) | 0.095 |
|  | | | | Anti Gram-positive agents | | | 77 (30.1) | 20 (19.8) | 0.064 |
|  | | | | Antifungals | | | 223 (87.1) | 80 (79.2) | 0.071 |
| Continuous data are presented with median (IQR) and categorical variables as count (%). | | | | | | | | | |
